# Heterogeneity of PD-L1 expression in primary tumors and paired lymph node metastases of triple negative breast cancer

**DOI:** 10.1186/s12885-017-3916-y

**Published:** 2018-01-02

**Authors:** Ming Li, Anqi Li, Shuling Zhou, Yan Xu, Yaoxing Xiao, Rui Bi, Wentao Yang

**Affiliations:** 1 Department of Pathology, Fudan University Shanghai Cancer Center, 270 Dongan Road, Shanghai, 200032 China; 2 Department of Oncology, Shanghai Medical College, Fudan University, Shanghai, People’s Republic of China

**Keywords:** Triple negative breast cancer, PD-L1, Lymph node metastasis, Heterogeneity

## Abstract

**Background:**

Programmed cell death ligand 1 (PD-L1) is a potential predictive biomarker of the response to anti-PD-L1/anti- programmed cell death 1 (PD-1) therapy in multiple cancers, including triple negative breast cancer(TNBC). The purpose of this study was to investigate whether PD-L1 expression is homogenous in primary tumors(PTs) and synchronous axillary lymph node metastases(LNMs) of TNBC.

**Methods:**

PD-L1 expression was immunohistochemically evaluated in 101 TNBC patients’ PTs and paired LNMs. PD-L1 expression in tumor cells and infiltrating immune cells or node lymphocytes in the PTs and associated LNMs was scored separately and was correlated with patients’ clinical parameters and prognoses.

**Results:**

PD-L1 expression exhibited spatial heterogeneity in both the tumor cells and the infiltrating immune cells or node lymphocytes of PTs and LNMs. The PD-L1 expression levels were significantly higher in the lymphocytes and tumor cells of the LNMs than in the PTs. PD-L1 expression was also more frequent among the LNMs. PD-L1 expression was associated with high grade and more stromal tumor-infiltrating lymphocytes(TILs). Furthermore, the disease-free survival and overall survival were similar between the PT- negative/LNM- positive and PT- positive/LNM- positive patients, both of which exhibited worse disease-free survival(DFS) thanPT -negative/LNM -negative patients.

**Conclusions:**

The differential expression of PD-L1 between the PTs and LNMs suggests that LNMs PD-L1 status may be used to indicate whether PD-1/PD-L1-targeted therapy would be suitable for a node-positive TNBC patient in the future.

**Electronic supplementary material:**

The online version of this article (10.1186/s12885-017-3916-y) contains supplementary material, which is available to authorized users.

## Background

Programmed cell death ligand 1 (PD-L1, also known as B7-H1 or CD274) is believed to mediate local immune evasion in many types of cancer by binding to programmed cell death 1 (PD-1), its co-stimulatory receptor on T cells, to induce saturation of activated anti-tumor T cells [1]. Recently, PD-1 and PD-L1 have been shown to be promising targets for the treatment of different tumor types [2]. In particular, triple negative breast cancer (TNBC) comprises 10–15% of all breast cancer cases and usually exhibits a poorer clinical prognosis than non-TNBC, as it appears to be an aggressive subtype of breast cancer and lacks therapeutic targets [3]. As previous studies showed that TNBC had more frequently PD-L1 expression [4, 5], anti-PD-L1/anti-PD-1 therapy has become a promising therapeutic strategy for TNBC, and several trials have shown that anti-PD-1 therapy was effective for breast cancer, and particularly TNBC [6, 7].

PD-L1 protein expression in tumor cells and infiltrating immune cells has been used as a biomarker to predict the responses of TNBC patients to anti-PD-L1/anti-PD-1 therapy [8]. However, certain patients with negative PD-L1 expression have been observed to respond to PD-1/PD-L1-blockade therapy [9]. The reason for this finding may be the dynamic nature of PD-L1 expression during the progression of breast cancer [10], as shown in a previous study demonstrating PD-L1 status conversion from negative in the primary tumor (PT) to positive in lung metastasis in 1 of 12 TNBC patients [11]. Therefore, exclusion of patients whose PTs exhibit negative PD-L1 expression from anti-PD-L1/anti-PD-1 therapy might omit potential responders.

Lymph nodes are the initial and the most frequent sites of breast cancer metastasis [12]; thus, lymph node metastasis (LNM) formation is a crucial step in breast cancer progression. Half of the primary TNBC exhibit lymph node involvement, and these patients have poorer prognoses than patients without lymph node involvement [13]. In terms of the important role of the PD-1/PD-L1 axis in immune system evasion [14], we hypothesized that PD-L1 expression would be more frequent and stronger in LNMs than in PTs.

Here, we aimed to elucidate the differences in PD-L1 expression between PTs and paired LNMs by examining the PD-L1 statuses of 101 node-positive TNBC patients’ PTs and synchronous axillary LNMs. In addition, we assessed the association between PD-L1 expression and the clinicopathological features as well as the prognosis of node-positive TNBC patients.

## Methods

### Sample selection

A total of 101 lymph node-positive TNBC patients who had received surgical treatment at Fudan University Shanghai Cancer Center from February 1, 2007 to December 31, 2011 and for whom resected PT and synchronous LNM tissues were available were consecutively retrieved from a pathology database. The patients were recruited according to the following criteria: (i) female gender; (ii) histologically confirmed invasive ductal carcinoma (IDC) with an ER-/PR-/HER2-negative phenotype, (iii) no evidence of distant metastasis at diagnosis, (iv) no receipt of any type of treatment prior to surgery, and (v) at least one tumor-positive axillary lymph node. The clinicopathological features of all patients were reviewed. The stromal tumor-infiltrating lymphocytes(TILs) was evaluated referred to the International TILs Working Group 2014 [15].

### Immunohistochemistry (IHC)

IHC was performed using 4-μm-thick sections of representative formalin-fixed PT and synchronous axillary LNM tissue blocks. Briefly, the slides were dewaxed in xylene, passed through graded alcohols, and placed into 0.01 mol/L phosphate-buffered saline (PBS; pH = 7.4). The slides were then pretreated with 1.0 mM citrate, pH 6.0 (Invitrogen), in a steam pressure cooker for epitope retrieval and were washed in PBS. Next, they were incubated with 3% hydrogen peroxide for 15 min to block endogenous peroxidase activity and were subsequently incubated with a monoclonal rabbit anti–human PD-L1 antibody (CST, 13,684, 1:150) at 4 °C overnight. The antibody was previously reported to have been used by Ali et al. for breast cancer tissue staining [16].On the following day, the slides were washed with PBS and incubated with an anti-rabbit secondary antibody (Dako) for 60 mins at room temperature. After being washed in PBS, the slides were stained with DAB+ (Dako) and then counterstained for 1 min with Harris hematoxylin (BASO), differentiated in 1% hydrochloric acid in alcohol, dehydrated, and mounted. A negative control was prepared by replacing the primary antibody with 0.1% bovine serum albumin (BSA). All PT and LNM specimens were stained using the same protocol. To validate the antibody, MDA-MB-231 cell line was treated with siRNA against PD-L1, and then assessed by western blot analysis (Additional file 1: Figure S1A).

### Evaluation of PD-L1 expression

PD-L1 expression was independently assessed by two experienced breast pathologists, AQL and YX, who had no prior knowledge of the patients’ clinical information. Tumor cells and infiltrating immune cells or nodal lymphocytes were scored separately in the PTs and the associated LNMs. Considering the spatial heterogeneity of PD-L1 expression [9, 17], we decided to focus on the hot spots in which PD-L1 staining was particularly prevalent. The percentage of PD-L1 expression was calculated by quantifying the total number of positive cells, as previously demonstrated in a recommendation evaluating Ki67 expression [18], with mandatory inclusion of all hot spots and the invasive edge of the tumor in the sections. The percentage of membranous PD-L1 expression was scored in 5% increments ranging from 0 to 100%, and a score of over 5% was considered to indicate PD-L1 positivity [19].

### Statistical analysis

Statistical analyses were performed using SPSS 20 statistical software. Correlations between PD-L1 expression in tumor cells and lymphocytes in the PTs and LNMs were examined using the Wilcoxon matched-pairs signed-rank test and Spearman’s rank correlation. Correlations between PD-L1 expression and the clinicopathological features of the TNBC patients were evaluated using the chi-squared test and Fisher’s exact test. Survival curves were plotted using the Kaplan-Meier method within GraphPad Prism 5.0. A *p*-value of less than 0.05 was considered statistically significant.

## Results

### Spatial heterogeneity of PD-L1

**Fig. 1:**
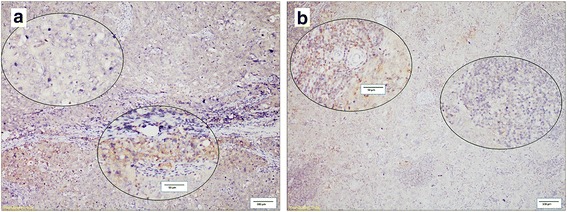
Heterogeneous staining of PD-L1. The two circled areas are shown at a higher magnification to illustrate PD-L1 heterogeneity and intra-tumoral expression in tumor infiltrating immune cells (**a**) and tumor cells (**b**). Scale bar = 100 μm, and scale bar of inset = 50 μm

PD-L1 is expressed in tumor cells and associated infiltrating immune cells or nodal lymphocytes, and its expression showed spatial heterogeneity in the PTs and LNMs in this study. PD-L1 expression was observed in the lymph node germinal centers, providing an internal positive control for staining (Additional file 1: Figure S1B). The expression displayed a multifocal distribution and was limited to the tumor-stroma interface in most PTs (Fig. 1a). Similar to what was observed in the PTs, PD-L1 was also expressed at the interface between lymphocytes and tumor cells in the LNMs (Fig. 1b).

### Discordance of PD-L1 expression between tumor cells and lymphocytes in the PTs and LNMs

**Fig. 2:**
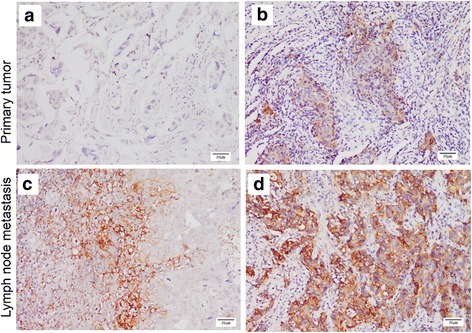
Differences in PD-L1 expression between PTs and LNMs. Case 1 showed negative PD-L1 expression in a PT (**a**) and positive expression in an LNM (**b**). Case 2 showed a PT exhibiting a low level of PD-L1 expression (**c**), and an LNM showing a moderate level of PD-L1 expression (**d**). Scale bar = 50 μm

Specimens in which PD-L1 expression was detected in tumor cells and/or lymphocytes were defined as PD-L1 positive. PD-L1 expression was identified in the PTs of 39 patients (38.61%). Among these 39 patients, 31 (30.69%) possessed PD-L1-positive infiltrating immune cells (range, 5–60%; median = 10%), and 26 (25.74%) had positive tumor cells (range, 5–70%; median = 15%). PD-L1 expression was more frequently observed in the LNMs (*p* < 0.0001),as it was detected in the LNMs of 60 patients (59.41%). Among these patients, 54 (53.46%) possessed positive nodal lymphocytes (range, 5–80%; median = 20%), and 41 (40.59%) had positive tumor cells (range, 5–80%; median = 10%). In summary, 21/101 (20.79%) exhibited negative PD-L1 expression in PTs but positive in the paired LNMs (Fig. 2).

**Fig. 3:**
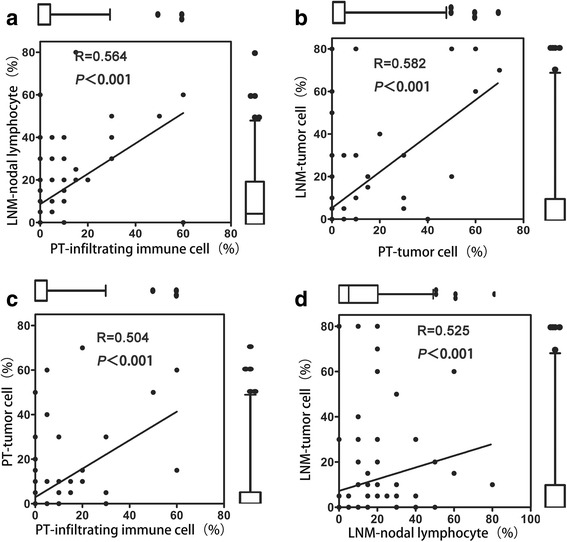
Comparison of the heterogeneity of PD-L1 expression between PTs and LNMs. The box plot shows the correlation between PD-L1 expression in tumor cells and infiltrating lymphocytes. The Wilcoxon signed-rank test for paired samples was performed to assess statistical significance. **a** Association between lymphocyte PD-L1 expression in matched PT and LNM specimens. Significantly higher expression was detected in the LNMs. **b** Association between tumor cell PD-L1 expression in matched PT and LNM specimens. Significantly higher expression was detected in the LNMs. **c** Correlation between PD-L1 expression in tumor cells and lymphocytes in PTs. No significant differences were observed. **d** Correlation between PD-L1 expression in tumor cells and lymphocytes in LNMs. No significant differences were observed

To determine the relationship between PD-L1 expression in PTs and LNMs, we examined the correlation between its expression in the matched specimens using Spearman’s rank correlation test. A moderate association between lymphocytes PD-L1 expression in the matched PT and LNM specimens was detected (*R* = 0.564, *p* < 0.001) (Fig. 3a), similar to what was observed in tumor cells (*R* = 0.582, *p* < 0.001) (Fig. 3b). Next, to investigate PD-L1 heterogeneity, we assessed the differences in PD-L1 expression between the primary and metastatic tissues using the Wilcoxon matched-pairs signed-rank test. The heterogeneity of lymphocyte PD-L1 expression significantly differed between the PTs and the LNMs (*p* < 0.001), as observed in tumor cells (*p* = 0.0051). These data suggested that PD-L1 expression in LNMs was stronger than in PTs.

PD-L1 expression between tumor cells and lymphocytes was significantly positively correlated in both the PTs (Spearman’s rank correlation = 0.504; *p* < 0.001) (Fig. 3c) and the LNMs (Spearman’s rank correlation = 0.525; *p* < 0.001) (Fig. 3d). In addition, the differences in PD-L1 expression between the lymphocytes and tumor cells in the PTs and LNMs were independently assessed using the Wilcoxon matched-pairs signed-rank test, and no significant differences were observed in either the PTs (*p* = 0.8192) or the LNMs (*p* = 0.1458).

### PD-L1 expression and clinicopathological features in matched PTs and LNMs

The associations of PD-L1 positivity with the variable clinicopathological features of PT-tumor cells, PT-infiltrating lymphocytes, LNM-tumor cells and LNM-lymphocytes are summarized in Table 1. The presence of PD-L1-positive infiltrating immune cells in the PTs was significantly associated with high histological grade(*p* = 0.031). The presence of PD-L1-positive infiltrating immune cells (*p* = 0.020) and tumor cells (*p* = 0.001) in the PTs was significantly associated with high TIL score. In addition, tumor cell PD-L1 expression in the LNMs was significantly associated with increased recurrence (*p* = 0.013). Lymphocytes PD-L1 expression in the LNMs was significantly associated with increased distant metastasis (*p* = 0.033). No significant relationships were observed between PD-L1 expression and patient age, menopausal status, the number of positive lymph nodes, or tumor size in the PT-tumor cells, PT-infiltrating lymphocytes, LNM-tumor cells or LNM-lymphocytes.

**Table 1:** Clinical characteristics in patients with tumor cell or lymphocyte PD-L1 expression

Variable	Overall		PD-L1											
			PT-Infiltrating immune cell(+)			PT-tumor cell (+)			LNM-lymphocyte(+)			LNM-tumor cell(+)		
	N	%	N	%	*p*	N	%	*p*	N	%	*p*	N	%	*p*
	101		31	30.69		26	25.74		54	53.46		41	40.59	
Age, years					0.667			0.823			0.841			0.543
≤ 50	45	44.55	15	48.39		11	42.31		25	46.30		20	48.78	
> 50	56	55.46	16	51.61		15	57.69		29	53.70		21	51.22	
Menopausal Status					0.661			1			0.689			0.410
Post	60	59.41	17	54.84		15	57.69		31	57.41		22	53.66	
Pre	41	40.59	14	45.16		11	42.31		23	42.59		19	46.34	
Tumor size					1			0.816			0.541			0.149
≤ 2 cm	38	37.62	12	38.71		9	34.62		22	40.74		19	46.34	
> 2 cm	63	62.38	19	61.29		17	65.38		32	59.26		22	53.66	
Histological grade					*0.031*			0.130			1			0.175
II	28	27.72	4	12.90		4	15.38		15	27.78		8	19.51	
III	73	72.28	27	87.10		22	84.62		39	72.22		33	80.49	
Node status					0.198			0.545			0.623			0.280
pN1 (1–3 LNs)	52	51.49	19	61.29		16	61.54		30	55.55		25	61.98	
pN2 (4–9 LNs)	29	28.71	9	29.03		6	23.08		15	27.78		10	24.39	
pN3 (≥10 LNs)	20	19.80	3	9.68		4	15.38		9	16.67		6	14.63	
TIL score(%)					*0.020*			*0.001*			0.399			*0.042*
0–10	60	59.41	12	38.71		7	26.92		32	59.26		21	51.22	
11–20	22	21.78	10	32.26		10	38.46		14	25.93		14	34.15	
≥ 21	19	18.81	9	29.03		9	34.62		8	14.81		6	14.63	
Local recurrence					0.506			0.072			0.134			*0.013*
absence	89	88.12	26	83.87		20	76.92		45	83.33		32	78.05	
presence	12	11.88	5	16.13		6	23.08		9	16.67		9	21.95	
Distant metastasis					0.797			0.592			*0.033*			0.094
absence	78	77.23	23	74.19		19	73.08		37	68.52		28	68.29	
Presence	23	22.77	8	25.81		7	26.92		17	31.48		13	31.71	

*Abbreviations*: *PD-L1* programmed cell death ligand 1, *PT* primary tumor, *LNM* lymph node metastasis, *TIL* tumor infiltrating lymphocyte

*p*-value of less than 0.05 was considered statistically significant

To evaluate the relationship between PD-L1 expression in PTs and LNMs, we combined the PT and LNM PD-L1 expression data and stratified all cases into three groups (PT negative/LNM negative (PT-/LNM-), PT negative/LNM positive (PT-/LNM+), and PT positive/LNM positive (PT+/LNM+)). In contrast, no significant clinicpathological differences were found among the three groups, except for differences in TIL score(*p* = 0.028) (Additional file 2: Table S1).

### Prognostic significance of PD-L1 expression in PTs and LNMs

**Fig. 4:**
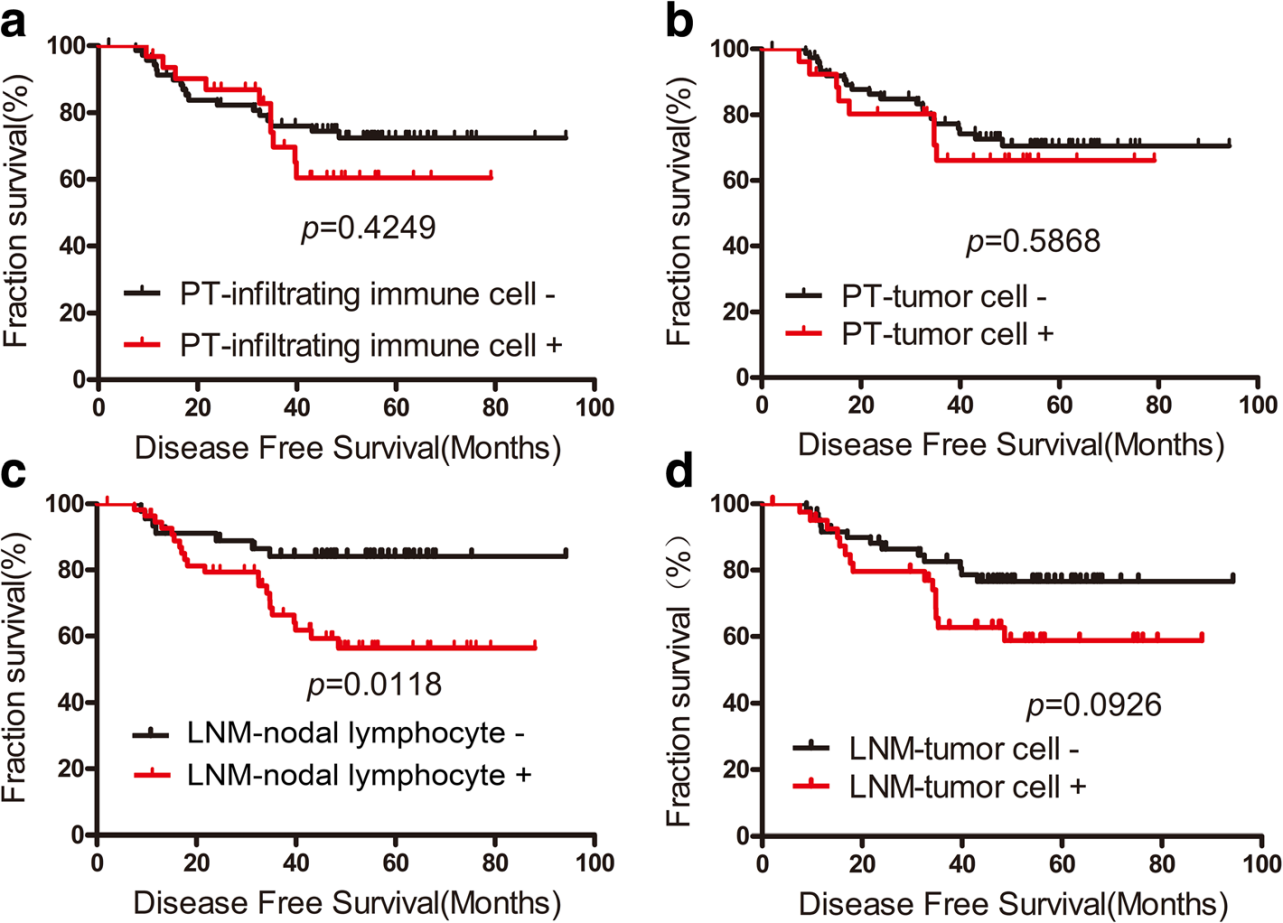
Kaplan–Meier survival curve for disease-free survival according to PD-L1 expression in PTs and LNMs. DFS was not significantly worse in patients with PD-L1 expression in the  PT and tumor cells in the LNM (**a**, **b**, **d**). DFS was significantly worse in patients with PD-L1 expression in the nodal lymphocytes (**c**)

We compared disease-free survival (DFS) and overall survival (OS) separately according to PD-L1 expression in tumor cells and infiltrating immune cells in the PTs and associated LNMs (Fig. 4 and Additional file 3: Figure S2). The median age at diagnosis was 51 years (range, 27–74 years), and the median follow-up time was 49.03 months (range, 10.97–94.27 months). Patients with PD-L1 expression in lymphocytes of LNM exhibited significantly worse DFS (HR = 2.598; 95% CI: 1.236–5.460; *p* = 0.0118). There was no significant association between DFS and PD-L1 expression in the PTs and tumor cell in the LNM. No significant association between PD-L1 expression and OS was observed.

**Fig. 5:**
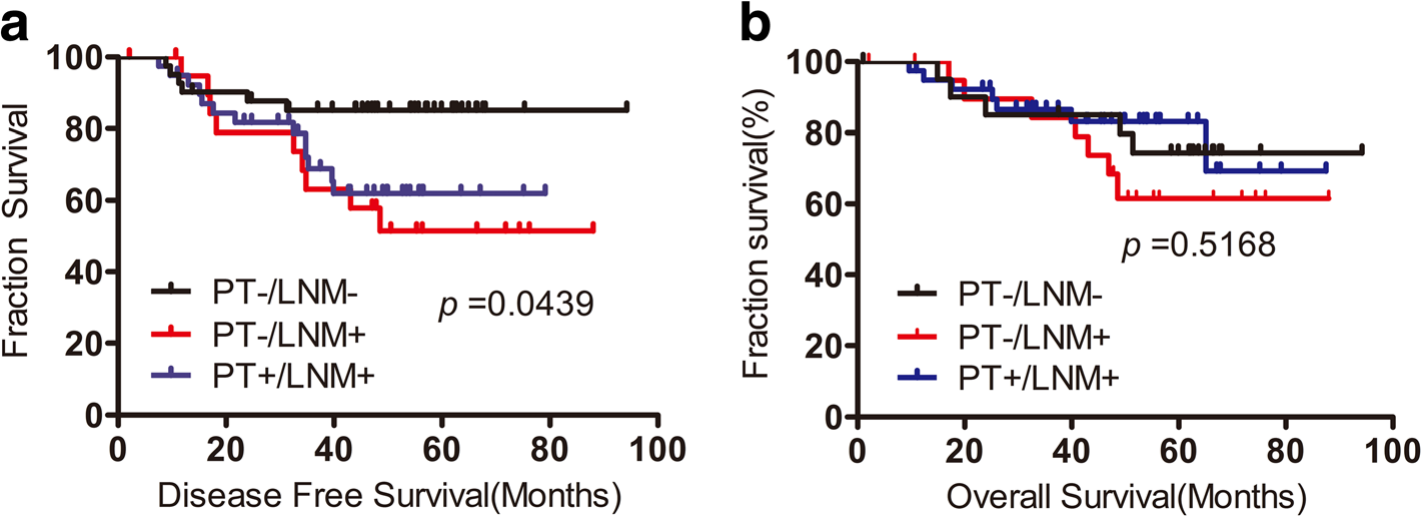
Kaplan–Meier survival curve for disease-free survival and overall survival according to PD-L1 expression. **a** DFS was significantly worse in PD-L1 PT-/LNM+ and PT+/LNM+ patients. **b** OS was not significantly different among three groups

The disease-free survival (DFS) rates significantly differed among the three groups of patients (PT-/LNM-, PT-/LNM+, and PT+/LNM+) (*p* = 0.0439) (Fig. 5). We also compared the DFS rates between pairs of groups and found that the PT-/LNM+ (HR = 3.824; 95% CI: 1.282–11.41; *p* = 0.0161) and PT+/LNM+ (HR = 2.487, 95% CI: 1.007–6.145; *p* = 0.0483) groups showed worse DFS than the PT-/LNM- group. Overall survival (OS) was also analyzed and was not found to significantly differ among the three groups (*p* = 0.5168) (Fig. 5, Additional file 4: Table S2). The multivariate prognostic analysis also indicated that PD-L1 expression in LNMs (HR = 2.92; 95% CI: 1.18–7.22; *p* = 0.02) and LN status (HR = 1.60; 95% CI: 1.02–2.52; p = 0.04) were independent factors for DFS (Additional file 5: Table S3).

## Discussion

This study revealed differences in PD-L1 expression between LNMs and paired PTs in both tumor cells and infiltrating immune cells or nodal lymphocytes in node-positive TNBC. Furthermore, the presence of PD-L1-positive tumor cells was significantly associated with a high score of TIL. PD-L1 expression was also associated with worse DFS, and the PT-/LNM+ and PT+/LNM+ groups had similar DFS rates. The results of this study suggest that testing of only PT specimens might result in exclusion of a potentially responsive subgroup of PT-/LNM+ patients from receiving anti-PD-L1/anti-PD-1 therapy.

PD-L1 expression in breast cancer has been frequently evaluated in recent studies, most of which have used tissue microarrays (TMAs) due to their large sample sizes and including a variety of breast cancer subtypes [4, 20]. Considering the spatial heterogeneity [9] of PD-L1 expression, we selected representative slides for evaluation by IHC, rather than using TMAs, and scored tumor cells and lymphocytes separately in PTs and paired LNMs. In our study, PD-L1 expression was detected in 38.61% (39/101) of the PTs of node-positive TNBC patients, with 31 (30.69%) exhibiting PD-L1-positive infiltrating lymphocytes, and 26 (25.74%) possessing positive tumor cells. PD-L1 expression was significantly associated with poorer survival. These results are relatively consistent with those of two recent studies showing that PD-L1 expression is a poor prognostic marker in breast cancer patients [4, 20]. One of these studies specifically reported the detection of PD-L1 expression in 59% of all TNBC cells; this rate was higher than that observed in the current study. However, another large-scale study using TMAs has shown that PD-L1 is expressed in 19% of basal-like tumors in association with improved disease-specific survival [16]. Other methods have also been used to evaluate PD-L1 expression. For example, one study measured PD-L1 expression using DNA microarrays, which revealed positive expression in 38% of basal tumors [5]. Another study using in situ mRNA hybridization coupled with TMAs detected PD-L1 mRNA expression in nearly 60% of breast cancer cells [21]. These two studies both demonstrated that PD-L1 expression is a good prognostic indicator. To date, however, no standardized assays have been developed for evaluation of tumor PD-L1 expression, as there are no specific anti-PD-L1 monoclonal antibodies available for use in IHC, no set criterion for a PD-L1 “positive” tumor, and no standard methods.

Differences in the levels of molecular markers, including ER, PR, HER2 [22] and epithelial-to-mesenchymal transition-related markers [23], between primary breast cancers and both lymph nodes and distant metastases have been frequently demonstrated in previous studies. These results suggest that making treatment decisions solely based on the expression of these molecular markers in PTs may result in the inappropriate use of hormone and targeted therapies in cancer patients. The heterogeneity of PD-L1 status has also been reported in clear cell renal cell carcinoma [24] and bladder cancer [25]. Our findings demonstrated that the paired LNMs (59.41%) more commonly and strongly exhibited PD-L1 expression than the PTs (38.61%) did, with 20.79% of the node-positive TNBC patients demonstrating negative-to-positive conversion of their PD-L1 status. Moreover, our results revealed that PT-/LNM+ patients showed worse DFS than the PT-/LNM- group and showed similar DFS with the PT+/LNM+ group. Thus, PD-L1 negativity in a PT may be not sufficient to exclude a node-positive TNBC patient from receiving anti-PD-L1 therapy. We postulate that measurement of PD-L1 expression in LNMs could improve the selection of patients for treatment by identifying an increased number of potential responders.

The discordance detected in PD-L1 expression between PTs and paired LNMs reflects the dynamic nature of this protein. Many hypotheses could explain this expression difference. First, many studies have demonstrated that PD-L1 expression is upregulated in tumor cells stimulated by inflammatory cytokines, and particularly interferons (IFNs) produced by infiltrating immune cells [1, 26]. In addition, one study has indicated that basal-like breast cancer cells have the capacity to evade the immune system via upregulation of PD-1 ligands adapted to IFN-c, which is secreted by T helper cells [10]. Thus, the enriched infiltrating T cells in lymph nodes may drive PD-L1 expression to induce adaptive immune resistance during infiltration of tumor cells [27]. Second, loss of PTEN expression is a mechanism that could regulate PD-L1 expression in TNBC patients [19], as has previously been described in glioma patients [28]. Clonal selection may be an additional mechanism that promotes discordance in PD-L1 expression between PTs and LNMs [29].

Further studies using a larger cohort of patients are warranted to confirm the differences in PD-L1 expression between PTs and LNMs in node-positive TNBC patients. Factors associated with the induction of local PD-L1 expression and conversion in LNMs should also be identified. Furthermore, tumor infiltrating immune cells and nodal lymphocyte subsets within tumor microenvironments in PTs and LNMs should be analyzed.

## Conclusion

In conclusion, we have demonstrated that LNMs have stronger and more frequent PD-L1 expression than paired PTs, suggesting that PTs are not adequate surrogates for determining PD-L1 expression in LNMs. We thus postulate that the measurement of PD-L1 expression in LNMs could increase the accuracy of predicting patient prognosis and better allow for optimal treatment selection.

## Additional files

Additional file 1: Figure S1. The validation of PD-L1 antibody. (A) Western blot analysis for PD-L1 using MDA-MB-231 treated with control and PD-L1 targeting siRNAs. (B) Immunoarchitecture of a TNBC lymph nodal metastasis. PD-L1 expression was observed in the lymph node germinal centers, providing an internal positive control for staining. (TIFF 6516 kb)

Additional file 2: Table S1. Clinicopathological features of the three groups: PT-/LNM-, PT-/LNM+ and PT+/LNM+ (DOCX 19 kb)

Additional file 3: Figure S2. Kaplan–Meier survival curve for overall survival (OS) according to PD-L1 expression in PTs and LNMs. (TIFF 5345 kb)

Additional file 4: Table S2. Cox regression analysis of PD-L1 expression and clinicopathological factors predicting OS. (DOCX 15 kb)

Additional file 5: Table S3. Cox regression analysis of PD-L1 expression and clinicopathological factors predicting DFS. (DOCX 15 kb)

## Abbreviations

BSA: Bovine serum albumin
DFS: Disease-free survival
IDC: Invasive ductal carcinoma
IHC: Immunohistochemistry
LNM: Lymph node metastasis
OS: Overall survival
PBS: Phosphate-buffered saline
PD-1: Programmed cell death 1
PD-L1: Programmed cell death ligand 1
PT: Primary tumor
TIL: Tumor-infiltrating lymphocyte
TMA: Tissue microarray
TNBC: Triple negative breast cancer
