# Supplementary figures and images for: Heterogeneity of PD-L1 expression in primary tumors and paired lymph node metastases of triple negative breast cancer
Source: BMC Cancer. 2018 Jan 2;18:4. doi: 10.1186/s12885-017-3916-y (PMC5748959; doi:10.1186/s12885-017-3916-y)

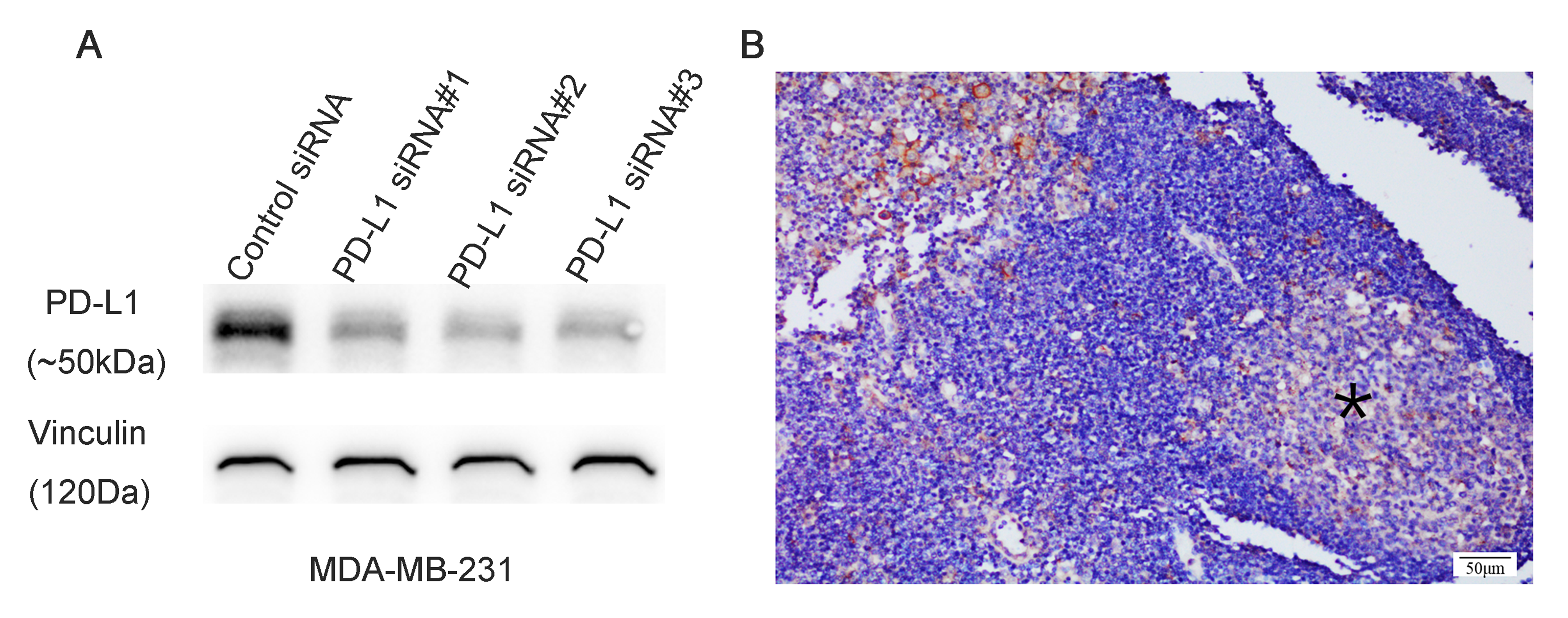

Supplement: Supplementary file 1 — The validation of PD-L1 antibody. (A) Western blot analysis for PD-L1 using MDA-MB-231 treated with control and PD-L1 targeting siRNAs. (B) Immunoarchitecture of a TNBC lymph nodal metastasis. PD-L1 expression was observed in the lymph node germinal centers, providing an internal positive control for staining. (TIFF 6516 kb) [file 12885_2017_3916_MOESM1_ESM.tif]

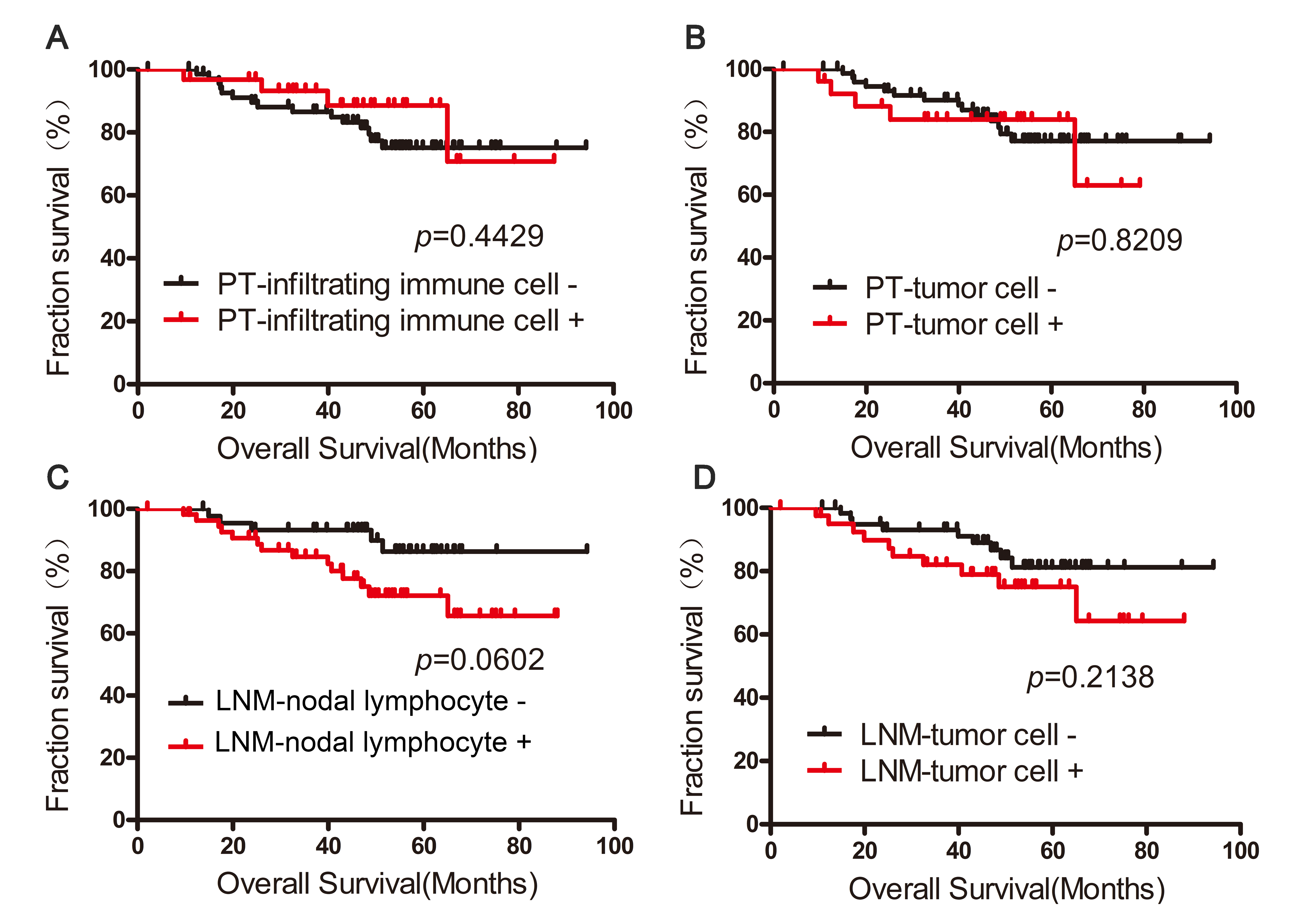

Supplement: Supplementary file 3 — Kaplan–Meier survival curve for overall survival (OS) according to PD-L1 expression in PTs and LNMs. (TIFF 5345 kb) [file 12885_2017_3916_MOESM3_ESM.tif]
