# Supplementary material for: Heterogeneity of PD-L1 expression in primary tumors and paired lymph node metastases of triple negative breast cancer
Source: BMC Cancer. 2018 Jan 2;18:4. doi: 10.1186/s12885-017-3916-y (PMC5748959; doi:10.1186/s12885-017-3916-y)
Supplement: Supplementary file 2 — Clinicopathological features of the three groups: PT-/LNM-, PT-/LNM+ and PT+/LNM+ (DOCX 19 kb) [file 12885_2017_3916_MOESM2_ESM.docx]

**Additional file 2: Table S1**

Clinicopathological features of the three groups: PT-/LNM-, PT-/LNM+ and PT+/LNM+

| Variable | PD-L1 | | | | | | |
| --- | --- | --- | --- | --- | --- | --- | --- |
|  | PT-/LNM- | | PT-/LNM+ | | PT+/LNM+ | | *p* |
|  | 41 | 40.59% | 21 | 20.79% | 39 | 38.61% |  |
| Age, years |  |  |  |  |  |  | 0.901 |
| ≤50 | 17 | 41.46% | 10 | 47.62% | 18 | 46.15% |  |
| ＞50 | 24 | 58.54% | 11 | 52.38% | 21 | 53.85% |  |
| Menopausal Status |  |  |  |  |  |  | 0.837 |
| Post | 26 | 63.41% | 12 | 57.14% | 22 | 56.41% |  |
| Pre | 15 | 36.59% | 9 | 42.86% | 17 | 43.59% |  |
| Tumor size |  |  |  |  |  |  | 0.602 |
| ≤2cm | 14 | 34.15% | 10 | 47.62% | 14 | 35.90% |  |
| ＞2cm | 27 | 65.85% | 11 | 52.38% | 25 | 64.10% |  |
| Histological grade |  |  |  |  |  |  | 0.059 |
| II | 13 | 31.71% | 9 | 42.86% | 6 | 15.38% |  |
| III | 28 | 68.29% | 12 | 57.14% | 33 | 84.62% |  |
| Node status |  |  |  |  |  |  | 0.426 |
| pN1 (1-3 LNs) | 18 | 43.90% | 10 | 47.62% | 24 | 61.54% |  |
| pN2 (4-9 LNs) | 14 | 34.15% | 5 | 23.81% | 10 | 25.64% |  |
| pN3 (≥10 LNs) | 9 | 21.95% | 6 | 28.57% | 5 | 12.82% |  |
| TIL score(%) |  |  |  |  |  |  |  |
| 0-10 | 27 | 65.85% | 17 | 80.95% | 16 | 41.03% | **0.028** |
| 11-20 | 8 | 19.51% | 3 | 14.29% | 11 | 28.20% |  |
| ≥21 | 6 | 14.63% | 1 | 4.76% | 12 | 30.77% |  |
| Local recurrence |  |  |  |  |  |  | 0.180 |
| absence | 39 | 95.12% | 18 | 85.71% | 32 | 82.05% |  |
| presence | 2 | 4.88% | 3 | 14.29% | 7 | 17.95% |  |
| Distant metastasis |  |  |  |  |  |  | 0.062 |
| absence | 36 | 87.80% | 13 | 61.90% | 29 | 74.36% |  |
| presence | 5 | 12.20% | 8 | 38.10% | 10 | 25.64% |  |
| Abbreviations: PD-L1, programmed cell death ligand 1; PT, primary tumor; LNM, lymph node metastasis; TIL, tumor infiltrating lymphocyte. | | | | | | | |
