# Supplementary material for: Heterogeneity of PD-L1 expression in primary tumors and paired lymph node metastases of triple negative breast cancer
Source: BMC Cancer. 2018 Jan 2;18:4. doi: 10.1186/s12885-017-3916-y (PMC5748959; doi:10.1186/s12885-017-3916-y)
Supplement: Supplementary file 4 — Cox regression analysis of PD-L1 expression and clinicopathological factors predicting OS. (DOCX 15 kb) [file 12885_2017_3916_MOESM4_ESM.docx]

Additional file 4: **Table S2** Cox regression analysis of PD-L1 expression and clinicopathological factors predicting OS

| Variable | Univariate analysis | | | | | Multivariate analysis | | | | |
| --- | --- | --- | --- | --- | --- | --- | --- | --- | --- | --- |
|  | | β | HR | 95% CI | *p* | | β | HR | 95% CI | *p* |
| PT-PD-L1 | | 0.017 | 1.02 | 0.40-2.59 | 0.97 | |  |  |  |  |
| LNM-PD-L1 | | 0.75 | 2.12 | 0.76-5.90 | 0.15 | |  |  |  |  |
| Age | | -0.26 | 0.77 | 0.31-1.91 | 0.58 | |  |  |  |  |
| Menopausal Status | | 0.01 | 1.01 | 0.40-2.51 | 0.99 | |  |  |  |  |
| Tumor size | | 0.86 | 2.37 | 1.06-5.27 | **0.04** | | 0.69 | 1.99 | 0.92-4.30 | 0.08 |
| Histological grade | | -0.15 | 0.86 | 0.33-2.26 | 0.76 | |  |  |  |  |
| Node status | | 0.66 | 1.94 | 1.11-3.37 | **0.02** | | 0.57 | 1.78 | 1.01-3.12 | **0.046** |
| TIL score | | -0.41 | 0.66 | 0.33-1.35 | 0.26 | |  |  |  |  |
| Abbreviations: PD-L1, programmed cell death ligand 1; DFS, disease-free survival; HR, hazard ratio; CI, confidence interval; PT, primary tumor; LNM, lymph node metastasis; TIL, tumor infiltrating lymphocyte. | | | | | | | | | | |
