# Supplementary material for: Heterogeneity of PD-L1 expression in primary tumors and paired lymph node metastases of triple negative breast cancer
Source: BMC Cancer. 2018 Jan 2;18:4. doi: 10.1186/s12885-017-3916-y (PMC5748959; doi:10.1186/s12885-017-3916-y)
Supplement: Supplementary file 5 — Cox regression analysis of PD-L1 expression and clinicopathological factors predicting DFS. (DOCX 15 kb) [file 12885_2017_3916_MOESM5_ESM.docx]

**Additional file 5: Table S3** Cox regression analysis of PD-L1 expression and clinicopathological factors predicting DFS

| Variable | Univariate analysis | | | | | Multivariate analysis | | | | | | |
| --- | --- | --- | --- | --- | --- | --- | --- | --- | --- | --- | --- | --- |
|  | | β | HR | 95% CI | *p* | | β | | HR | | 95% CI | *p* |
| PT-PD-L1 | | 0.42 | 1.53 | 0.73-3.21 | 0.27 | |  | |  | |  |  |
| LNM-PD-L1 | | 1.06 | 2.90 | 1.17-7.16 | **0.02** | | 1.07 | | 2.92 | | 1.18-7.22 | **0.02** |
| Age | | -0.38 | 0.68 | 0.33-1.44 | 0.32 | |  | |  | |  |  |
| Menopausal Status | | -0.23 | 0.80 | 0.38-1.67 | 0.54 | |  |  | | |  |  |
| Tumor size | | 0.49 | 1.63 | 0.84-3.15 | 0.15 | |  |  | | |  |  |
| Histological grade | | -0.19 | 0.83 | 0.38-1.84 | 0.65 | |  | |  |  | |  |
| Node status | | 0.48 | 1.61 | 1.01-2.57 | **0.045** | | 0.47 | | 1.60 | | 1.02-2.52 | **0.04** |
| TIL score | | -0.17 | 0.84 | 0.50-1.41 | 0.51 | |  | |  | |  |  |
| Abbreviations: PD-L1, programmed cell death ligand 1; DFS, disease-free survival; HR, hazard ratio; CI, confidence interval; PT, primary tumor; LNM, lymph node metastasis; TIL, tumor infiltrating lymphocyte. | | | | | | | | | | | | |

.
